# Exploiting chitosan and gold nanoparticles for antimycobacterial activity of *in silico* identified antimicrobial motif of human neutrophil peptide-1

**DOI:** 10.1038/s41598-019-44256-6

**Published:** 2019-05-27

**Authors:** Richa Sharma, Ragini Raghav, Kumari Priyanka, Praveen Rishi, Sadhna Sharma, Sudha Srivastava, Indu Verma

**Affiliations:** 1 Department of Biochemistry, Post Graduate Institute of Medical Education and Research, Chandigarh, India; 2 Department of Biotechnology, Jaypee Institute of Information Technology, Noida, Uttar Pradesh India; 3 Department of Microbiology, Panjab University, Chandigarh, India; 4 Present Address: Albert Einstein College of Medicine, Bronx, New York USA

**Keywords:** Nanoparticles, Peptide delivery, Antibiotics

## Abstract

The upsurge of drug resistant tuberculosis is major health threat globally. To counteract, antimicrobial peptides are being explored as possible alternatives. However, certain limitations of peptide-based drugs such as potential toxicity, high cost and relatively low stability need to be addressed to enhance their clinical applicability. Use of computer predicted short active motifs of AMPs along with nanotechnology could not only overcome the limitations of AMPs but also potentiate their antimicrobial activity. Therefore, present study was proposed to in silico identify short antimicrobial motif (Pep-H) of human neutrophil peptide-1 (HNP-1) and explore its antimycobacterial activity in free form and using nanoparticles-based delivery systems. Based on colony forming unit analysis, motif Pep-H led to killing of more than 90% *M. tb in vitro* at 10 μg/ml, whereas, similar activity against intracellularly growing *M. tb* was observed at 5 μg/ml only. Thereafter, chitosan (244 nm) and gold nanoparticles (20 nm) were prepared for Pep-H with both the formulations showing minimal effects on the viability of human monocyte derived macrophages (MDMs) and RBC integrity. The antimycobacterial activity of Pep-H against intracellular mycobacteria was enhanced in both the nanoformulations as evident by significant reduction in CFU (>90%) at 5–10 times lower concentrations than that observed for free Pep-H. Thus, Pep-H is an effective antimycobacterial motif of HNP-1 and its activity is further enhanced by chitosan and gold nanoformulations.

## Introduction

Antimicrobial peptides (AMPs) have great potential to be explored as efficient drug candidates against tuberculosis (TB), the leading cause of deaths amongst infectious diseases across the globe^1^. The complex survival strategies employed by mycobacteria to reside successfully inside the host led to usage of lengthy multidrug cocktail regime for more than forty years. Lack of addition of new drugs to the age old therapy and patient incompliance has provided favourable conditions for mycobacteria to evolve into various drug resistant strains and posing great challenges for TB control. Thus, AMPs, essential part of our first line innate immune defense, having ability to not only form cytotoxic pores in bacterial membranes, but also inhibit cell wall, nucleic acid, and protein biosynthesis are appropriate contenders to enhance efficacy of current chemotherapy.

Previously our laboratory reported Human neutrophil peptide-1 (HNP-1), a human α-defensin (AMP), as a potential adjunct to existing chemotherapy and also established its specific mode of action against mycobacteria^2–4^. However, there are various drawbacks of a peptide drug to be used as a pharmacological agent. The problems that hamper the progress of AMPs as clinical candidates include lack of stability, short *in vivo* half-life, proteolytic cleavage, toxicity and high cost of manufacturing. Recently, studies have focused on exploring the role of shorter peptides of AMPs as ideal therapeutic candidates against range of infectious agents^5^. These shorter peptides are more economical, easier and faster to synthesize, less immunogenic and can be easily tuned to minimize toxicity, enhance stability and specificity, increase half-life^5,6^. Unique features constituting pharmacophore of AMPs that facilitate their interaction with pathogenic membranes include cationic charge provided by Arg and Lys, amphipathic nature and α-helical structure. Various in silico prediction tools have also emerged which exploit different properties of these AMPs to generate prediction algorithms that are helpful in identifying such short pharmacophore of any AMP^7,8^.

Another approach to overcome the limitations of AMPs based therapy is to utilize nanotechnology for safer delivery and improved activity. The smaller size and high drug loading capacity of nanoparticles enable them to deliver drugs safely by crossing various cellular barriers^9^. Peptide therapeutics can be encapsulated and conjugated on different types of polymeric or metallic nanoparticles for their better applicability^10^. Amongst various polymers used for nanoformulations, chitosan, an abundant natural polymer is biocompatible, non-toxic and biodegradable^11^. Various recent reports have indicated chitosan as a promising candidate for encapsulation of antimicrobial peptides against various infections. Chitosan nanoparticles loaded with the antimicrobial peptide temporin B have been reported to exert effective antibacterial activity *in vitro* against clinical isolates of *Staphylococcus epidermidis*^11^. Similarly, nano-encapsulated cryptdin formulation against *Salmonella* infection had shown improved oral therapeutic potential with prolonged and continuous release of peptide in intestinal mucosa^12^. In case of metallic nanoformulations, gold nanoparticles have been considered as most promising for drug delivery due to their small size, high solubility, stability, biocompatibility and chemical inertness^13^. Moreover, large surface-area-to-volume ratio in gold nanoparticles, allows the adsorption of several hundred molecules on its surface^14^. There are very limited studies available on the use of AMPs loaded gold nanoparticles^15,16^. More importantly, the potential of nanoformulations of AMPs against mycobacterial infection is as yet unexplored. Present study was therefore designed to identify functional pharmacophore of HNP-1 to evaluate its activity against *Mycobacterium tuberculosis* (*M. tb*) using chitosan and gold nanoparticles.

## Results

### Selection of HNP-1 motif

The web servers generated 15 amino acid long peptide fragments of HNP-1 and antimicrobial scores were designated for each fragment to predict their antimicrobial activity. Based on the positive charge and antimicrobial scores obtained, the functional motif was selected. Out of 16 peptide fragments of HNP-1, RRYGTCIYQGRLWAF, was predicted commonly as antimicrobial fragment by all the four servers obtaining antimicrobial scores of 0.806, 0.089, 0.946 and 0.218 along with highest positive charge (+3) amongst all the fragments (Table 1). Thus, peptide RRYGTCIYQGRLWAF, named as Pep-H was selected as antimicrobial motif of HNP-1. Pep-H was further subjected to *in vitro* and *ex vivo* assays to establish its antimycobacterial efficacy.

**Table 1:** Antimicrobial scores of short peptides of HNP-1 obtained from AntiBP, AntiBP2, CAMP and AMPA servers.

Peptide fragments	Position	Antimicrobial Score				Charge	*Antimicrobial motif
		AntiBP	AntiBP2	CAMP	AMPA		
ACYCRIPACIAGERR	1–15	**1.001**	**−**0.454	**0.662**	—	+2	Yes
CYCRIPACIAGERRY	2–16	**1.120**	**−**0.702	**0.816**	—	+2	Yes
YCRIPACIAGERRYG	3–17	**−**0.276	**−**0.566	**0.656**	—	+2	NO
CRIPACIAGERRYGT	4–18	**1.080**	**−**1.048	0.377	—	+2	NO
RIPACIAGERRYGTC	5–19	**0.833**	**−**0.249	**0.682**	—	+2	Yes
IPACIAGERRYGTCI	6–20	**−**1.425	**−**0.629	0.296	—	+1	NO
PACIAGERRYGTCIY	7–21	**−**0.261	**−**0.996	**0.505**	—	+1	NO
ACIAGERRYGTCIYQ	8–22	**0.829**	**0.637**	**0.519**	—	+1	Yes
CIAGERRYGTCIYQG	9–23	**−**0.515	**−**0.051	0.450	—	+1	Yes
IAGERRYGTCIYQGR	10–24	**−**0.368	**−**0.242	0.357	—	+2	NO
AGERRYGTCIYQGRL	11–25	**0.652**	**0.151**	**0.662**	—	+2	Yes
GERRYGTCIYQGRLW	12–26	**−**0.643	**−**1.066	**0.885**	—	+2	NO
ERRYGTCIYQGRLWA	13–27	−0.593	**−**0.513	**0.955**	—	+2	NO
RRYGTCIYQGRLWAF*	**14–28**	**0.806**	**0.089**	**0.946**	**0.218**	+**3**	**Yes**
RYGTCIYQGRLWAFC	15–29	**−**0.302	**−**0.467	**0.840**	—	+2	NO
YGTCIYQGRLWAFCC	16–30	**−**0.447	**−**0.287	0.325	—	+1	NO

The peptide highlighted in bold has antimicrobial potential based on antimicrobial score predictions by respective servers. *Antimicrobial motif: a short peptide having highest positive charge and predicted as antimicrobial based on scores obtained from all the servers. The motif RRYGTCIYQGRLWAF (highlighted in box) was selected as motif based on its antimicrobial score predictions.

### Activity of Pep-H against *in vitro* growing *M. tb* H37Rv

**Figure 1:**
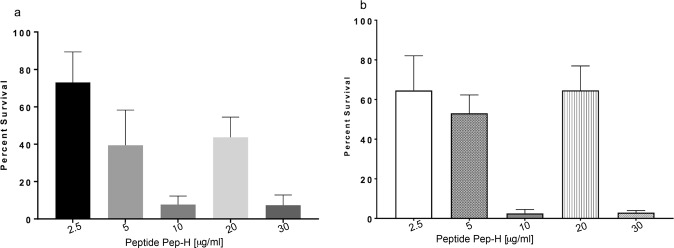
Activity of antimicrobial motif Pep-H against *M. tb* H37Rv. Mycobacterial cells were grown *in vitro* in the presence of different concentrations of Pep-H for 7 days and the percentage of viable bacilli left after the treatment was determined based on (**a**) absorbance measurements and (**b**) CFU enumeration. Percent survival was calculated as OD or CFU (Treated)/OD or CFU (Control)*100. Values are expressed as Mean ± S.D. of 3–4 independent experiments. For control (Untreated *M. tb* H37Rv), values were considered as reported earlier^18^.

Log phase *M. tb* was grown in the presence of different concentrations of Pep-H for 7 days to determine anti-mycobacterial potential of Pep-H by monitoring absorbance and colony forming units (CFU). Based on absorbance measurements, nearly 40% and 8% bacteria survived after treatment with Pep-H at 5 μg/ml and 10 μg/ml respectively (Fig. 1a). This indicated that Pep-H displayed 60% and 92% inhibitory activity against *M. tb* H37Rv at 5 μg/ml and 10 μg/ml respectively and 10 μg/ml was taken as its MIC^17^. These results were further confirmed by CFU enumeration that depicted the bactericidal activity of Pep-H at 10 μg/ml against *M. tb* as only 3% mycobacterial growth was observed as compared to control (Fig. 1b).

### Effect of Pep-H against intracellular *M. tb* H37Rv

**Figure 2:**
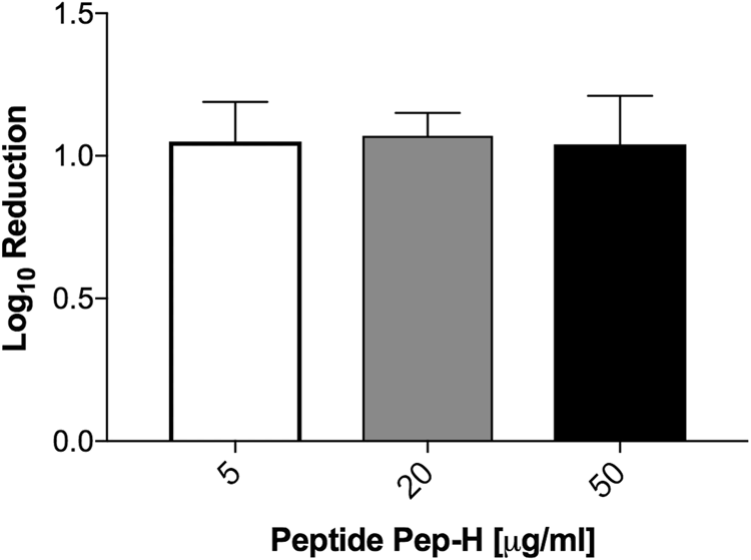
Activity of antimicrobial motif Pep-H on the growth of *M. tb* H37Rv inside MDMs. For antimycobacterial activity of Pep-H against intracellular bacilli, MDMs were infected with *M. tb* H37Rv followed by incubation with different concentrations of Pep-H. Reduction in intracellular bacterial load based on CFUs is presented as Log10 reduction as Mean ± SD of three independent experiments. Log10 reduction is calculated as Log_10_CFU (treated)-Log_10_CFU(Control)/Log_10_CFU(Control). The Log10CFU/ml in control (untreated infected MDMs) was 5.79 ± 0.08 as reported earlier^18^.

Since *M. tb* is an intracellular pathogen, it is important to monitor the activity of any therapeutic candidate against *M. tb* multiplying inside the host cells. In the present study, PBMCs isolated from the blood of healthy volunteers were differentiated into macrophages and the effect of Pep-H on the growth of *M. tb* H37Rv replicating in MDMs was monitored in terms of its bactericidal activity by CFU analysis. Pep-H showed 1 log (91%) reduction at 5 μg/ml in intracellular mycobacterial growth (Fig. 2). No further increase in the bactericidal activity of Pep-H was observed at higher concentrations. The inhibitory potential of Pep-H against intracellular *M. tb* was comparable to that obtained with anti-tubercular drugs rifampicin and isoniazid at 5 μg/ml and 3 μg/ml respectively as reported earlier by our group^18^. The increase in growth inhibition from 60% (against *in vitro M. tb*) to 91% (against intracellular *M. tb*) at the concentration 5 μg/ml indicates the possibility of immunomodulation of host cells by Pep-H as AMPs are well known to have immune-stimulatory effect. Therefore, cytokine levels in culture supernatants from Pep-H treated and control infected MDMs were estimated.

**Figure 3:**
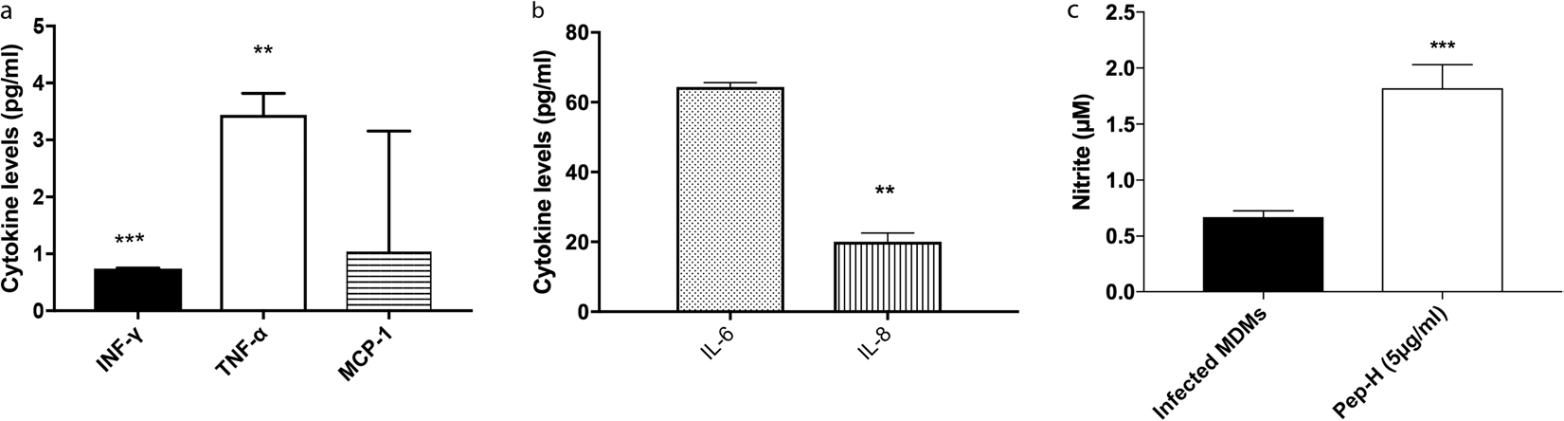
Levels of (**a**) IFN-γ, TNF-α, MCP-1, (**b**) IL-6, IL-8 and (**c**) NO released by *M. tb* H37Rv infected MDMs after treatment with Pep-H. Values are expressed as Mean ± SD of three independent experiments. The values of IFN-γ, TNF-α, MCP-1, IL-6, IL-8 obtained in control untreated infected MDMs are 0.15 ± 0.02, 41 ± 15.22, 2.05 ± 0.81, 231.53 ± 85.33, 40.23 ± 2.36 respectively as reported earlier^18^. For statistical analysis, one-way ANOVA was used and comparison was done with the levels of cytokines obtained in untreated infected MDMs. ***p < 0.001, **p < 0.01, w.r.t Control (Untreated infected MDMs).

Estimation of immune effectors indicated that Pep-H led to significant increase in the levels of IFN-γ and RNOS as compared to control infected MDMs (Fig. 3a,c). Pep-H treatment resulted in significant decrease in the levels of pro-inflammatory cytokines TNF-α (3.44 ± 0.38 pg/ml), IL-6 (64.35 ± 0.12 pg/ml) and MCP-1 (1.04 ± 2.12 pg/ml) (Fig. 3a,b). The increase in the levels of IFN-γ and RNOS following Pep-H treatment could be playing protective role along with direct anti-mycobacterial activity in restricting the growth of intracellular *M. tb*.

### Characterization of Pep-H loaded chitosan nanoparticles (Pep-H-CSNPs)

**Figure 4:**
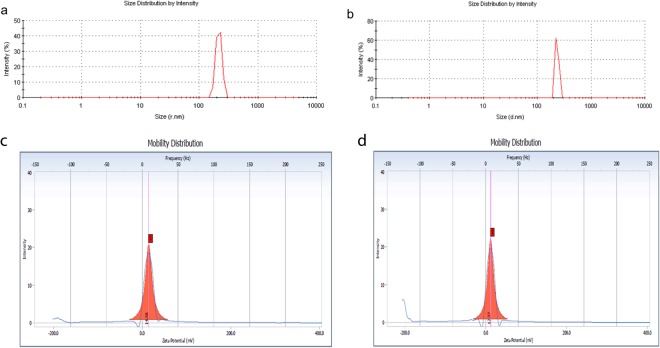
Representative graphs of particle size distribution (by intensity) of (**a**) CSNPs, (**b**) Pep-H-CSNPs and Zeta Potential of (**c**) CSNPs, (**d**) Pep-H-CSNPs. DLS was carried out with three different batches of chitosan nanoparticles and values shown are representative of one batch.

**Figure 5:**
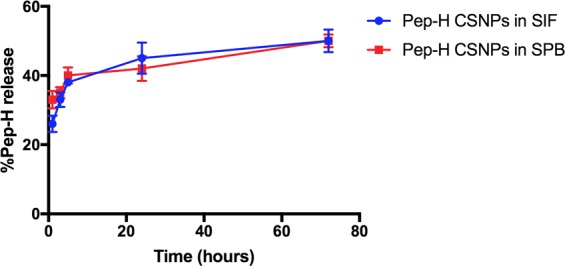
*In vitro* release profile of Pep-H loaded CSNPs in simulated intestinal fluid (SIF) and phosphate buffer (SPB). Chitosan nanoparticles (CSNPs/Pep-H-CSNPs) were placed in 1 ml SIF and phosphate buffer. After 1 hr, 3 hrs, 6 hrs, 24 hrs and 72 hrs, nanoparticles were pelleted down and the protein content was estimated in supernatants of all time points by BCA protein estimation assay. Values are expressed as Mean ± SD of three independent experiments.

The chitosan nanoparticles were prepared by simple process of ionic gelation, by dropwise addition of chitosan solution to TPP in the ratio of 5:2. The dynamic light scattering showed that average hydrodynamic size of Pep-H-CSNPs (244 ± 6 nm) was more as compared to empty CSNPs (197 ± 16 nm) (Fig. 4a,b). The polydispersity index of 0.16 indicated that the size of Pep-H-CSNPs formulations was uniformly distributed (Table 2). The zeta potential of Pep-H loaded nanoparticles was observed to be in range of +12 mV which suggested that the nanoparticles were positively charged and had less tendency to aggregate and flocculate (Fig. 4c,d). Further, narrow size distribution range with positive zeta potential indicates that Pep-H-CSNPs were stable and monodispersed. The amount of Pep-H encapsulated in CSNPs as determined by estimating the concentration of peptide in the supernatants showed entrapment efficiency to be 72% for Pep-H. The loading efficiency i.e. amount of peptide entrapped per mg of nanoparticles was 3.7% (Table 2). The release of Pep-H from Pep-H-CSNPs in simulated intestinal fluid (SIF) (pH 6.8) was examined for 72 hrs. The results showed initial rapid release of upto 30% peptide within 1 hr followed by a linear increase pattern. Thereafter, sustained release of 50% peptide was observed upto 72hrs in SIF. (Fig. 5). The release of Pep-H from Pep-H-CSNPs monitored in phosphate buffer (PB) (pH 7.4) for 72 hrs showed an early rapid release of around 40% with, a sustained release of 50% peptide till 3 days of incubation.

**Table 2:** The characteristics of chitosan nanoparticles (CSNPs) and Pep-H loaded chitosan nanoparticles (Pep-H-CSNPs) based on average size (nm), polydispersity index (PdI), Zeta potential (mV), encapsulation efficiency (%), and loading efficiency (%) and Yield (%).

Nanoformulations	Average Hydrodynamic Size (nm)	Polydispersity index	Zeta Potential (mV)	Encapsulation efficiency^a^ (%)	Loading efficiency^b^ (%)
CSNPs	197 ± 16	0.21 ± 0.03	+14.4 ± 0.16	—	—
Pep-H-CSNPs	244 ± 6	0.16 ± 0.06	+12.4 ± 0.33	72 ± 7.48	3.7 ± 1.06

Values are expressed as Mean ± SD of three independent batches of nanoparticles. ^a^Encapsulation efficiency is defined as the amount of peptide entrapped in nanoparticles per total peptide amount used. ^b^Loading efficiency is defined as the amount of peptide entrapped per mg of nanoparticle weight.

### Characterization of Pep-H conjugated gold nanoparticles (Pep-H-AuNPs)

**Figure 6:**
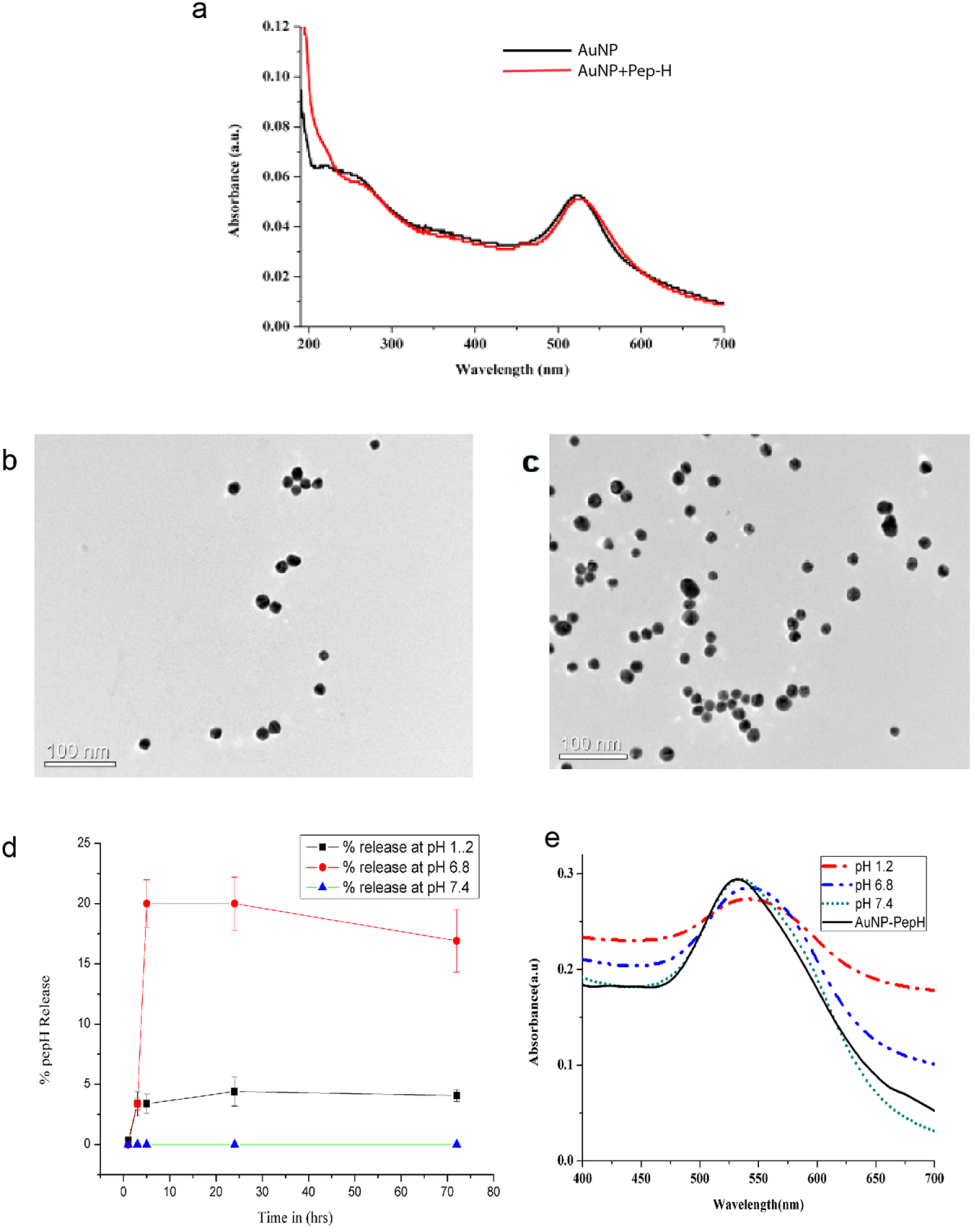
Characterization of Pep-H loaded AuNPs. (**a**) Absorption spectra of AuNPs and Pep-H-AuNPs, (**b,c**) TEM micrographs of AuNPs and Pep-H-AuNPs respectively for which AuNPs were coated on carbon grids and viewed under transmission electron microscope at magnification of 2,50,000x and scale of 100 nm (**d**) *In vitro* release profile of Pep-H-AuNPs in simulated intestinal fluid (SIF), simulated gastric fluid (SGF) and phosphate buffer (PB) for which gold nanoparticles (AuNPs/Pep-H-AuNPs) were placed in 1 ml SIF (pH-6.8)/SGF (pH1.2)/phosphate buffer (pH 7.4). After 1 hr, 3 hrs, 5 hrs, 24 hrs and 72 hrs, nanoparticles were pelleted down and the protein content was estimated in supernatants of all time points by Bradford assay (**e**) Stability of Pep-H-AuNPs as monitored by UV-Vis spectroscopy for any change in spectra of the pellets of Pep-H-AuNPs following incubation in SIF/SGF/PB. Values are expressed as Mean ± S.D. of 3 independent experiments.

The carboxyl group functionalized gold nanoparticles (AuNPs) were synthesized using aspartic acid as a reducing and capping agent via chemical reduction for tagging of Pep-H peptide on the surface. AuNPs exhibit a characteristic optical feature of surface plasmon resonance (SPR). The absorption spectra of gold nanoparticles (Fig. 6a) showed the absorption maxima at 523 nm that exhibits a red shift to 526 nm for Pep-H-AuNPs due to increased size and change in surface plasmon resonance (SPR). Presence of an additional hump around 220 nm, characteristic of peptide bond, confirms the conjugation of Pep-H with AuNPs.

Further, Pep-H-AuNPs were spherical in shape, with smooth surface morphology as observed by TEM (Fig. 6b,c). The size of AuNPs was analysed by measuring diameter of 20 nanoparticles per field at 3 equidistant points and 3 different fields were scanned per sample. The average size thus obtained for AuNPs was 15 ± 2 nm whereas that of Pep-H-AuNPs was 20 ± 4 nm. In a preparation of 4.7 × 10^10^ gold nanoparticles using 15 µg of Pep-H peptide, the amount of peptide on each nanoparticle was calculated to be 0.313fg which corresponded to 1 × 10^5^ peptide (Mw-1.89 kDa) molecules per AuNP. Percent release studies as measured by peptide estimation indicated no peptide release in the supernatant of the Pep-H-AuNPs in PBS at pH 7.4 even after 72 hrs of incubation. While in simulated gastric (SGF; pH 1.2) and simulated intestinal (pH 6.8) fluids, a small amount of peptide was released only after 3 hrs of incubation with marginal increase upto 5 hrs beyond which remained constant till 72 hrs. In SIF, the peptide released was in the range of 18–20% whereas in SGF, it was only 3–5% (Fig. 6d).

Further, absorption spectra of corresponding supernatants up to 1 hr of incubation of Pep-H-AuNPs conjugate in SGF, SIF and PBS buffers, is marked by absence of characteristic peak of peptide bond (207, 214, 220 nm) or aromatic amino acid (280 nm), thus indicating that Pep-H–AuNPs bond is not broken (data not shown). Spectra of pellets of Pep-H-AuNPs obtained after incubation with different pH media exhibited no shift in SPR peak (at pH 7.4) with a minor red shift at pH 6.8 indicative of increased size. Further, spectral features with well-defined peak representative of dispersed particles (no agglomeration) suggested the stability of Pep-H-AuNPs at pH 7.4 and pH 6.8 (Fig. 6e). However, at pH 1.2, in addition to the red shift of absorption peak, there is substantial broadening of the peak as well suggestive of increased size and/or agglomeration of the particles (Fig. 6e).

### Uptake of Pep-H-AuNPs by MDMs

**Figure 7:**
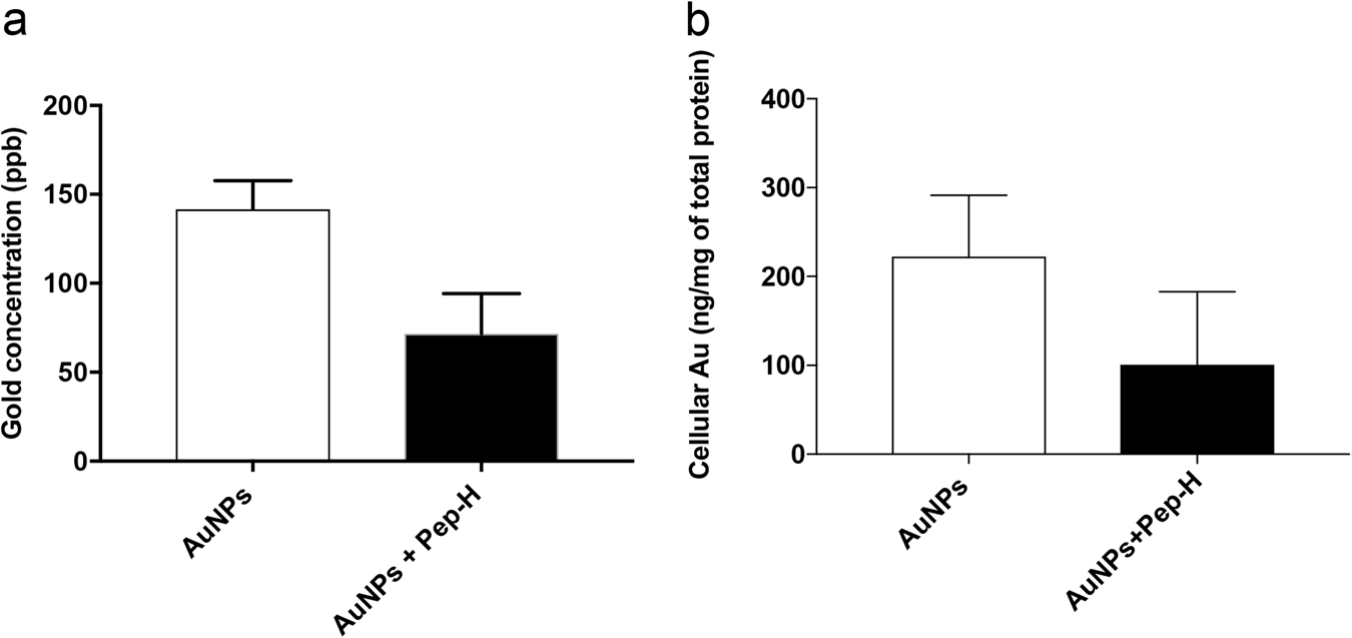
Uptake of Pep-H-AuNPs by MDMs. (**a**) The concentration of gold (ppb) measured in MDM monolayers after incubation of AuNPs and Pep-H-AuNPs after 24 hours. (**b**) The amount of gold accumulated inside the MDM monolayers after 24 hrs was expressed as amount of gold (ng) per mg of total cellular protein. Values are expressed as Mean ± SD of 3 independent experiments.

The gold nanoparticles are vehicles for intracellular drug delivery. Therefore, ability of Pep-H-AuNPs to permeate MDMs was determined. In order to estimate gold content inside the cells, monolayers incubated with AuNPs and Pep-H-AuNPs were subjected to Inductively Coupled Plasma Mass Spectrometry (ICP MS). The measured cellular mean gold content (ppb) was 141.65 ± 16.06 and 71.82 ± 22.50 for AuNPs and Pep-H-AuNPs respectively (Fig. 7a). The amount of gold taken up by MDMs was also normalized by the amount of cellular protein in each sample lysate and accumulation was expressed as amount of gold per unit mass of protein as presented in Fig. 7b. Although, uptake of nanoparticles based on amount of gold per unit mass of protein was lesser in case of Pep-H-AuNPs, however there was no significant difference as compared to AuNPs. These results indicated the uptake of both AuNPs and Pep-H-AuNPs by MDMs.

### Assessment of cytotoxicity of nanoformulations

**Figure 8:**
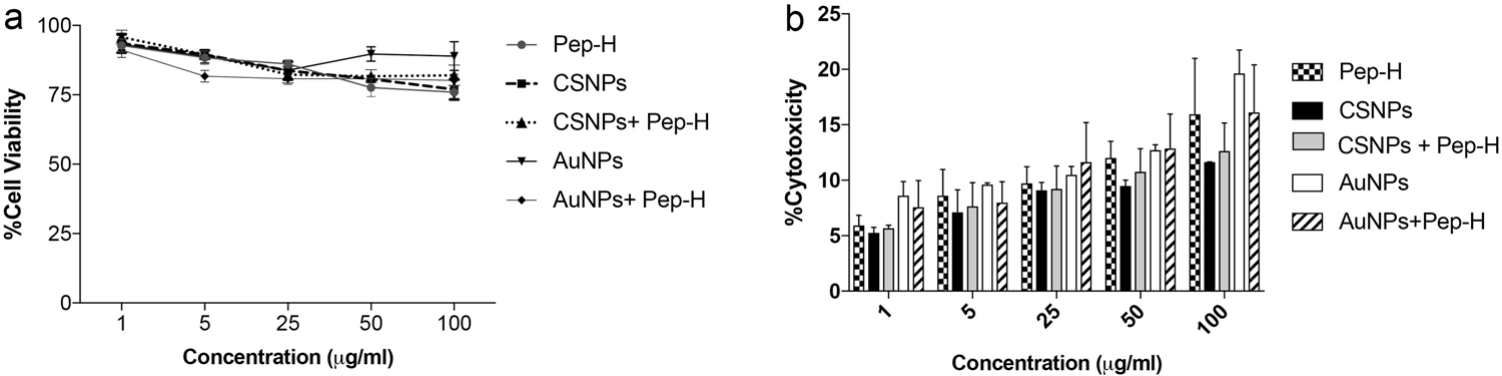
Evaluation of safety profile of Pep-H loaded nanoformulations against MDMs. (**a**) Percentage cell viability of MDMs upon treatment with different concentrations of Pep-H and its nanoformulations. (**b**) Percent cytotoxicity of Pep-H and its nanoformulations at different concentrations in MDMs. The cells were treated with various concentrations of Pep-H as well as Pep-H-CSNPs and Pep-H-AuNPs respectively for 72 hrs. The effect of this treatment on cell viability was assessed by MTT assay. The supernatants of these treated cells were subjected to LDH assay to determine cytotoxicity of free peptides and their nanoparticle formulations. Values are expressed as Mean ± S.D. of 3 independent experiments.

The effect of Pep-H and Pep-H nanoformulations on cell viability was evaluated by MTT assay performed in human MDMs. As evident from Fig. 8a, the optimum cell viability was seen for free Pep-H at 5 μg/ml whereas, at higher concentration of 100 μg/ml cell viability was observed to be 75%. In case of Pep-H-CSNPs and Pep-H-AuNPs also, more than 80% MDMs were viable at all the concentrations tested (Fig. 8a). These results were further supported by LDH release observed in culture supernatants to determine cytotoxicity of free peptides and nanoparticle formulations which indicated less than 20% cell cytotoxicity with increasing concentration up to 100 μg/ml for free Pep-H as well as peptide in nanoformulations (Fig. 8b). These results confirm the cytocompatibility of chitosan/gold nanoformulations used in the present study.

### Assessment of RBC integrity by hemolytic assay

**Figure 9:**
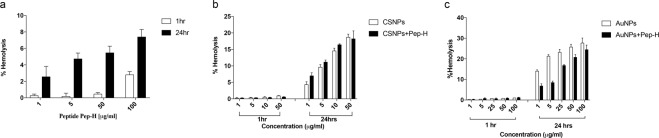
Stability of RBCs after treatment with Pep-H and its chitosan and gold nanoformulations. (**a**) Percent hemolysis exhibited by RBCs after incubating with different concentrations of Pep-H. (**b**,**c**) Percent hemolysis exhibited by RBCs after incubating with different concentrations of Pep-H-CSNPs and Pep-H-AuNPs respectively. The cells were treated with various concentrations of Pep-H as well as its nanoformulations respectively for 1 hr and 24 hrs and hemoglobin release was measured to determine the effect on RBC integrity. Values are expressed as Mean ± S.D. of 3 independent experiments.

Another important parameter for determining the toxicity of experimental therapeutic agents is the effect on RBC integrity. Incubation of RBCs isolated from healthy blood with increasing concentrations of Pep-H showed only 7% RBC lysis even after 24 hrs of treatment with Pep-H (100 μg/ml), a concentration 20 times higher than its MIC (Fig. 9a). Similarly, Pep-H-CSNPs displayed negligible haemoglobin release after 1 hr treatment but upto 21% RBC lysis was observed after 24 hrs with increasing concentration upto 50μg/ml as shown in Fig. 9b. Again, in case of AuNPs and Pep-H-AuNPs, very low haemoglobin release was exhibited after 1 hr but as incubation time was increased to 24 hrs 27% lysis of RBCs was observed when concentration was increased up to 100 μg/ml (Fig. 9c).

### Activity of Pep-H loaded CSNPs/AuNPs against *M. tb* H37Rv growing inside MDMs

**Figure 10:**
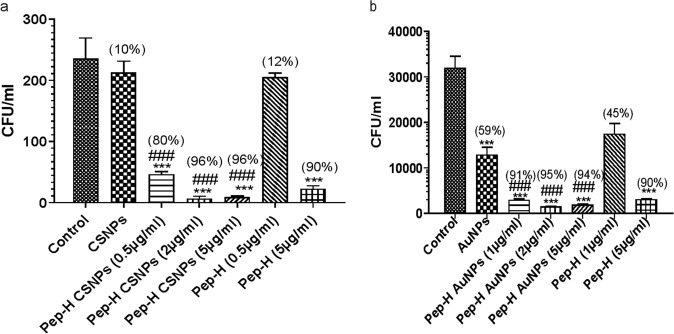
The antimycobacterial effect of (**a**) Pep-H-CSNPs and (**b**) Pep-H-AuNPs as compared to free Pep-H on *M. tb* H37Rv growing in the MDMs from healthy subjects. The results are represented as Mean CFU ± SD per ml of three independent experiments. Statistical analysis of the results was performed by one-way ANOVA. ***p < 0.001 w.r.t Control (untreated MDMs), ^###^p < 0.001 w.r.t CSNPs/AuNPs. Values in round brackets indicate percent inhibition obtained after respective treatments. Percent inhibition is calculated as CFU (Control) − CFU (treated)/CFU (Control) × 100.

Based on CFU enumeration, the antimycobacterial activity of Pep-H-CSNPs and Pep-H-AuNPs was observed to be markedly increased even at lower concentrations as compared to free Pep-H against *M. tb* H37Rv replicating inside MDMs (Fig. 10a,b). At 0.5 µg/ml, Pep-H-CSNPs resulted in 80% reduction in bacterial load as compared to only 12% reduction by free peptide (0.5 µg/ml). In case of Pep-H-AuNPs (1 µg/ml), 91% decrease in intracellular mycobacterial load was observed as compared to only 45% decrease by free Pep-H (1 µg/ml) treatment.

## Discussion

Tuberculosis is one of the major global health threats. It is highly needed to update chemotherapy regimen to control the spread of the disease efficiently. The tough mycobacterial cell wall makes it difficult for drugs to permeabilize and act on the pathogen. Antimicrobial peptides are known to act on microbial cell walls by creating pores, thus making them interesting candidates to explore for therapeutic potential. In our previous studies, HNP-1 has shown immense potential as anti-mycobacterial candidate. Currently the focus is on developing short peptide motifs of HNP-1, as they are easy to synthesize and easily modifiable to enhance antimicrobial potential and reduce toxicity. Various in silico tools employing different strategies for prediction of antimicrobial peptides such as using antimicrobial index of each amino acid^19^ and recognizing pattern of amino acids at N and C terminal in antimicrobial peptides^20,21^ have been exploited. Recently our group reported that Pep-B, the motif of HBD-1 selected using in silico prediction servers similar to present study demonstrated potential antimycobacterial activity against *in vitro* active as well as dormant tubercle bacilli^18^. By using similar in silico approach, we have identified short amino acid stretch, RRYGTCIYQGRLWAF (Pep-H) having highest positive charge as potential antimicrobial motif of HNP-1. Another study reported that this amino acid stretch forming conserved γ-core motif is a structural determinant of antimicrobial activity of α-defensins^22^. The motif Pep-H in the present study was observed to kill the *in vitro* growing *M. tb* but at higher concentration as compared to the earlier reported MIC of HNP-1^4^. The Pep-H peptide was used as such without any amino acid substitution whereas earlier by using various computational prediction servers, naturally occurring antimicrobial peptides have been modified by amino-acid substitution leading to antimicrobial potential even better than their parent peptide. hBD3-Pep4, a short decapeptide of HBD-3 designed by amino acid substitutions has been recently reported to inhibit the growth of *M. smegmatis*, at a concentration higher than MIC of HBD-3^23^. But a short truncated peptide of LL-37 substituted with positively charge amino acids was able to effectively kill *M. smegmatis* and *M. bovis* BCG by three fold as compared to LL-37 at the same concentration^24^. The in silico developed peptides, MIAP (300 μg/ml) and LLAP (600 μg/ml) have shown strong antimycobacterial activity as compared to magainin I (1200 μg/ml) and LL 37 (2400 μg/ml) against non-virulent strains of *M. tb*^7,8^. Noteworthy, as compared to earlier reported customised motifs, Pep-H without any modification in its sequence has resulted in to >90% killing of virulent mycobacteria at much lower concentration (10 μg/ml). Although, there was increase in the antimycobacterial activity of Pep-H with increasing concentration ranging from 0.5 μg/ml to 30 μg/ml, surprisingly, at 20 μg/ml, higher survival of mycobacterial cells was seen as compared to 10 μg/ml, the MIC of Pep-H and this was reproducibly observed in all the experiments. Although, the reason for the same is not understood, however, this kind of deviation at one or two concentrations while carrying out antimicrobial assays has also been observed in earlier studies. A report on the effect of different concentrations of Althaea officinalis extract on Lactobacillus acidophilus in terms of inhibitory growth zone diameter has indicated that from 3% to 50% concentration of the extract, there is gradual increase in the mean zone of microbial growth inhibition, whereas at 75%, there is again decrease in the growth inhibition followed by increase in inhibition at 100%^25^. Similar kind of deviation in the antibacterial activity curve in terms of log CFU/ml has been reported while investigating the effect of lecithin and eugenol against *E. coli*^26^.

*M. tb* is an intracellular pathogen that replicates efficiently in macrophages. It is important for therapeutic candidates to have ability to surpass this physical barrier so as to reach its target. PBMCs derived macrophages were used as *ex vivo* model to test the antimycobacterial activity of Pep-H. Pep-H showed marked reduction in intracellular mycobacterial growth at two times less concentration than in case of *in vitro* growing mycobacteria. This substantial decrease in mycobacterial growth was not due to any cytotoxic effect of the peptides on host cells. In contrast, hBD3-Pep4, LL-37 and its motif LLKKK-18 led to only 50–60% decrease in survival of *M. smegmatis* inside murine and human macrophage cell lines at higher concentrations than the peptide used in present study^23,24^. However, in earlier studies, the activity of AMPs has been explored against intracellular mycobacteria using various cell lines in contrast to human MDMs used in the current study. Recently, Pep-B motif of HBD-1 was also observed to result into >90% decrease in mycobacterial growth as compared to control infected MDMs^18^. In another study using human MDMs, LL-37 restricted the intracellular growth of *M. tb* in a dose dependant manner^27^.

Apart from direct antimicrobial role, AMPs are also known to modulate host immune system to help in the containment of infection. There are various studies showing immunomodulatory role of AMPs resulting into upregulated secretion of pro-inflammatory cytokines and chemokines. TNF-α and CXCL8 release in THP-1 and primary human macrophages by HBD-2 and HBD-3^28^, modulated levels of TNF-α, IL-17, IFN-γ, IL-10, TGF-β by LL-37 in *M. tb* infected macrophages^27^ and increased production of cytokines TNF-α, IL-6, and IL-12p70 in dendritic cells by HNP-1^29^ support the immunomodulatory role of AMPs. In a recent study, immunomodulatory potential of antimicrobial motif Pep-B of HBD-1 was observed^18^. Pep-H also led to significantly high release of IFN-γ and NO, however as compared to HNP-1 (data not shown), it significantly lowered the levels of proinflammatory cytokines including TNF-α, IL-6, IL-8 and MCP-1 suggesting that Pep-H is able to exert the antimycobacterial activity maintaining a balanced host inflammatory response.

Nanoscience has emerged as powerful technique for drug delivery and designing effective nano-delivery vehicles would help in enhancement of clinical applicability of AMPs as drugs. Although various nanoformulations have been exploited for antimicrobial activities of AMPs against various pathogens^11,12,30–32^, however, their utility for boosting the therapeutic potential of AMPs as drugs against mycobacteria is still unexplored. Both chitosan and gold have inherent antimicrobial and biodegradable properties^33–36^ and gold-based materials also exhibit interaction with immune system^37,38^. These features make them useful candidates to be explored for the development of therapeutic nanoformulations for AMPs against mycobacterial infections. The Pep-H-CSNPs had average diameter of 244 nm with uniform size distribution and stability with slow and constant release rate of peptide in SIF and phosphate buffer. There are very few reports available showing the successful encapsulation AMPs in polymer-based nanoparticles. Rishi *et al*., (2015) have shown the encapsulation of cryptidin in CSNPs with an average particle size of 105 nm^12^. Another study has shown encapsulation of temporin B in CSNPs having mean diameter of 185 nm, peptide entrapment of 75% along with linear and slow release of temporin in phosphate buffer^11^. The same authors have also reported the encapsulation of lysozyme in CSNPs, having mean size of 159 nm and positive surface charge^28^. In case of gold nanoparticles, capping agents such as surfactants play main role in stabilizing nanoparticles but create safety issues also. However, in present study amino acid Asp is used as a reducing and capping reagent for gold nanoparticles synthesis resulting in stable and biocompatible formulation. The –COOH group (Asp on gold nanoparticles) was exploited to form –CO-NH- linkage with -NH2 group of Arg located at N terminal of Pep-H and it was confirmed by red shift of 2–3 nm in the absorption spectra after peptide coating^39,40^. Further, stability studies indicated the stable complex of Pep-H-AuNPs at different pH corresponding to different physiological buffers. Minimal peptide release from Pep-H-AuNPs complex suggested that observed antimycobacterial activity in the present study was due to complex of gold with Pep-H as a whole. Another characteristic of nanoparticles is their ability to permeate the cells in order to deliver the drug intracellularly. This feature is particularly of great significance for drug delivery against intracellular pathogens like *M. tb*. Earlier, the uptake of FITC labelled CSNPs has been reported in J774.1, Caco-2 and HeLa cells^41,42^. In the present study also, uptake of CSNPs by MDMs was observed by incubating the rhodamine loaded CSNPs with macrophages (data not shown). In case of gold nanoparticles, uptake of both empty as well as Pep-H conjugated AuNPs by MDMs was observed. Earlier, several studies have reported the uptake of AuNPs with various cellular systems^43–45^. Various blood and cytocompatability assays confirmed biocompatibility of both chitosan and gold nanoformulations of Pep-H. Various studies have shown low hemolytic and negligible cytotoxic effect after exposure with CSNPs^11,46,47^. As compared to chitosan, the chemical inertness of gold accounts for its safety and gold-based compounds have been used clinically also as anti-inflammatory agents to treat rheumatoid arthritis^48^. There are sufficient reports indicating the safety profile of AuNPs in terms of negligible to very low cytotoxicity to various host cells/cell lines and hemolysis^43–45^. A recent study has reported that coating of amino acids on the surface of AuNPs minimizes the chance of coming in contact with RBCs, hence results in reduced hemolysis^49^ thus further supporting non-toxic and biocompatible nature of nanoformulations of Pep-H.

Along with their safety profile, both Pep-H nanoformulations led to marked increase in efficacy of Pep-H against intracellular mycobacteria at 5–10 times lower concentration than free Pep-H. Our results are supported by other studies that reported the effect of antimicrobial activity of AMPs like cryptidin, temporin, plectasin loaded in chitosan and PLGA nanoparticles against Salmonella and Staphylococcus strains^11,12,31^. In case of gold nanoparticles also, Pep-H-AuNPs using 1 μg/ml of Pep-H showed similar antimycobacterial activity as that shown by free Pep-H at 5 μg/ml. Interestingly AuNPs without Pep-H also showed significant decrease in intracellular bacterial CFU as compared to control infected MDMs, however antimycobacterial activity of Pep-H-AuNPs was significantly higher than that exhibited by AuNPs. Earlier, the antimicrobial activity of both gold and silver nanoparticles has been reported against various gram negative and gram positive bacterial pathogens^36,50^. The results of present study clearly demonstrate the combinatorial antimycobacterial effect of AuNPs and Pep-H exhibited by Pep-H-AuNPs as a complex rather than free peptide released from the gold nanoparticles. This was also supported by stability and peptide release assay indicating either no or minimal peptide release. There are studies showing the effect of different types of gold and silver nanoparticles against intracellular mycobacteria^51,52^, however, to the best of our knowledge, this is first report showing the effect of AMP loaded AuNPs against *M. tb*. Overall, the results of this study suggest that nanoformulations using chitosan or gold can be effectively used for the delivery of AMPs and could be explored further for the development of peptide based anti-TB therapeutic candidates.

In conclusion, this study highlights the therapeutic potential of an *in silico* predicted antimicrobial motif (Pep-H) from HNP-1, a human antimicrobial peptide, against *M. tb* in various nanoformulations.

## Methods

### *In silico* screening of antimicrobial motif of HNP-1

The amino acid sequence of HNP-1 was scanned using web servers AntiBP (www.imtech.res.in/raghava/antibp), AntiBP2 (www.imtech.res.in/raghava/antibp2/), AMPA (http://tcoffee.crg.cat/apps/ampa) and CAMP (http://www.camp.bicnirrh.res.in/) to identify an antimicrobial motif. These *in silico* tools generated antimicrobial scores based on different algorithms and these scores were used to identify a possible antimicrobial motif in a given protein. The antimicrobial motif thus selected was named as Pep-H and was got synthesized with >95% purity by RP-HPLC commercially from G.L. Biochem Ltd., China. The peptide was dissolved in 0.01% acetic acid and aliquots were frozen until further use at −80 °C.

### Mycobacterial culture

*Mycobacterium tuberculosis* (*M. tb*) H37Rv originally obtained from National Collection of Type Culture (NCTC), London and being maintained in the laboratory was used in this study. The culture was grown under continuous shaking in Sauton’s medium containing ADC and 0.05% Tween 80 at 37 °C and 200 rpm^53^. All experiments involving use of *M. tb* H37Rv strain were performed in Biosafety level 3 facility of the Institute.

### Activity of Pep-H against M. tb H37Rv growing *in vitro*

The antimycobacterial potential of Pep-H was determined by the broth-dilution method^2^. In brief, 1.5 × 10^8^ bacilli/ml from log phase culture were dispensed in 5 ml Sauton’s media along with Pep-H in the range of 1 µg/ml–40 µg/ml and incubated under continuous shaking for 7 days at 37 °C. To determine the minimal inhibitory concentration (MIC) (more than 90% reduction of growth), absorbance was measured at 620 nm^1^ and to determine the bactericidal activity, appropriate dilutions were plated on middlebrook 7H11 agar medium supplemented with OADC and colony-forming units (CFU) were enumerated after 3–4 weeks.

### Culture and maintenance of monocyte derived macrophages (MDMs)

This study protocol was approved by Institutional Ethics Committee, Post Graduate Institute of Medical Education and Research, Chandigarh, India. Informed consent was taken from all healthy participants before collecting blood samples. The methods were performed as per the relevant guidelines and regulations of Institutional Ethics Committee.

The heparinized blood was layered on Ficoll-hypaque and density gradient separation was done at 400 × g for 30 min to obtain peripheral blood mononuclear cells (PBMCs). PBMCs (2 × 10^6^ cells/ml) thus obtained were allowed to adhere overnight at 37 °C, 5% CO_2_ and were further cultured for 5–7 days to allow for maturation of monocytes into macrophages.

### Activity of Pep-H against intracellular *M. tb* H37Rv

Monocyte derived macrophages (MDMs) were dispensed (2 × 10^6^ cells/ml) in a flat bottom 24 well tissue culture plate and infected with *M. tuberculosis* H37Rv in the ratio of monocytes: mycobacteria::1:10 for 2 hrs. In order to remove extracellular non-phagocytosed mycobacteria, infected monolayer was washed thrice with RPMI-1640 without antibiotics and then media containing 2% FBS and amikacin (25 µg/ml) was added in each well and incubated for 2 hrs at 37 °C in 5% CO_2._ Thereafter infected monolayers were treated with different concentrations of Pep-H and cells were incubated for 72 hrs at 37 °C in 5% CO_2_. At the end of incubation, supernatants were removed and stored for cytokine analysis. Monolayer was washed gently with RPMI media and lysed with 500 μl of chilled 0.01% SDS. Lysates were appropriately diluted and plated on middle brook 7H11 agar medium supplemented with OADC and plates were incubated at 37 °C for 3–4 weeks. Colonies were counted and results were expressed as log_10_ CFU.

### Estimation of cytokines and reactive nitrogen species (RNOS) levels

Apart from direct antimycobacterial activity, effect of Pep-H on host immune system was also evaluated. To measure cytokines levels in the respective culture supernatants, BD Cytometric Bead Array (CBA) kit was used. IL-8 and MCP-1 concentrations were measured using anti human IL-8 and MCP-1 reagents (OptEIATM, BD Pharmingen, USA). Nitrite levels were determined by the Griess assay^54^.

### Formulation of Chitosan nanoparticles (CSNPs)

Chitosan (Sigma) was dissolved in 1% acetic acid (1 mg/ml; pH 5) under continuous stirring overnight. In order to load Pep-H, 100 μg of peptide was added to the chitosan solution and kept on stirring for 1 hr. Then sodium tripolyphosphate (TPP; Sigma) solution (1 mg/ml in water) was added dropwise through 26 gauge needle to chitosan solution kept under continuous stirring at the ratio of 5:2. The nanoparticles (Pep-H-CSNPs) formed spontaneously were kept on stirring for 2 hrs at room temperature and were separated by centrifugation at 22000 g for 1 hr at 4 °C followed by washing thrice^11^.

### Particle size and surface charge

The hydrodynamic particle size and polydispersity of freshly prepared CSNPs and Pep-H-CSNPs was measured by means of dynamic light scattering using Zetasizer (Malvern instruments, UK). For measuring the surface charge and stability of the formulation, Zeta potential was evaluated using particle size analyzer (Beckman-Coultier Delsa Nano C). Chitosan nanoparticles were dissolved in distilled water, sonicated for 5 min. and were passed through 0.45 μm sized syringe filters. The analysis was performed at 25 °C with angle of detection at 90 °C and dispersant refractive index and viscosity was selected as 1.33 and 0.88 for water respectively. The values thus obtained were average of analysis of three independent batches, each of them measured five times.

### Entrapment efficiency

The supernatants obtained after purification of nanoparticle suspensions of Pep-H-CSNPs by centrifugation were used for peptide estimation. The peptide content present in the supernatants was estimated by BCA protein assay Kit. As per manufacturer’s instructions, to estimate the peptide concentration in range of (5 μg/ml–250 μg/ml), samples and working solution were incubated at 60 °C for 30 min and optical density was measured at 562 nm.

The encapsulation efficiency was defined as the amount of peptide Pep-H recovered in the supernatant of Pep-H loaded nanoparticles (Pep-H-CSNPs) compared to the total amount of peptide used in the formulation and was calculated by following formula:

$${\rm{Encapsulation}}\,{\rm{efficiency}}=\frac{{\rm{Total}}\,{\rm{amount}}\,{\rm{of}}\,{\rm{peptide}}-{\rm{Amount}}\,{\rm{of}}\,{\rm{peptide}}\,{\rm{in}}\,{\rm{supernatant}}\times 100}{{\rm{Total}}\,{\rm{amount}}\,{\rm{of}}\,{\rm{peptide}}\,{\rm{added}}}$$

The loading efficiency was defined as the amount of peptide entrapped per mg of nanoparticles and was calculated as:

$${\rm{Loading}}\,{\rm{efficiency}}=\frac{{\rm{Total}}\,{\rm{amount}}\,{\rm{of}}\,{\rm{peptide}}-{\rm{Amount}}\,{\rm{of}}\,{\rm{unbound}}\,{\rm{peptide}}\times 100}{{\rm{Weight}}\,{\rm{of}}\,{\rm{Nanoparticles}}}$$

### *In vitro* peptide release kinetics of Pep-H-CSNPs

A suitable amount of peptide loaded nanoparticles equivalent to 15 μg of peptide were re-dispersed in 1 ml simulated intestinal fluid (pH 6.8) and 1 ml phosphate Buffer (pH 7.4) and kept at 37 °C under continuous stirring^11,12,55^. Nanoparticles were centrifuged at 22000 g for 30 min. at different time intervals (1 hr, 3 hr, 5 hr, 24 hr, 72 hrs). Supernatants were collected and the amount of peptide released was evaluated by modified BCA protein assay as explained above.

### Formulation of gold nanoparticles (AuNPs)

For synthesis of AuNPs, 3 ml of aspartic acid (100 mM) was added at a constant flow rate (0.1 ml/min.) to the boiling chloroauric acid. Heating and stirring was continued until the solution turned bright red and then it was quenched using an ice bath. Thereafter, sonication was done for 15 min. followed by centrifugation at 9000 rpm for 30 minutes. The pellet thus obtained consisted of the carboxyl functionalized AuNPs and was kept at room temperature till further processing^56^.

### Loading of Pep-H on AuNPs (Pep-H-AuNPs)

For loading of peptide on AuNPs, 100 μl of AuNPs were diluted in 50 ml MilliQ water and known amount of the peptide (1 μg/100 μl, 5 μg/100 μl, 10 μg/100 μl and 30 μg/100 μl) was added to the solution followed by incubation for 30 minutes at room temperature. The nanoparticles were collected by centrifugation at 9000 rpm for 30 minutes and pellet thus obtained was resuspended in 100 μl of MilliQ water and stored at 4 °C until further use. In order to use this formulation in *ex vivo* experiments, they were filter sterilized by passing through 0.2 μm syringe filters.

### Particle size and surface morphology

For particle size determination, 10 μl of sample (AuNPs/Pep-H-AuNPs) was placed onto carbon-coated copper grid, air dried and viewed under transmission electron microscope. The colloidal solution of gold nanoparticles was characterized by optical studies by comparing the UV-Vis spectra of AuNPs and Pep-H-AuNPs in the wavelength range of 400–900 nm.

### Estimation of number of peptide molecules on the surface of AuNPs

Using Beer lamberts Law with molar coefficient of extinction of 20 nm diameter AuNP as 8.8 × 10^8^ M^−1^ cm^−1^ and path length of 1 cm, concentration of AuNPs in colloidal solution was estimated to be 7.95 × 10^−10^ M which corresponded to 4.7 × 10^11^ gold nanoparticles/ml. Using 100 µL of AuNPs suspension and 15 µg of peptide, the average amount of peptide per nanoparticle was calculated.

### Stability of Pep-H-AuNPs and *in vitro* peptide release assay

A suitable amount of Pep-H-AuNPs equivalent to 15 μg of Pep-H were incubated in different pH media including simulated intestinal fluid (SIF) (pH 6.8), simulated gastric fluid (SGF) (pH 1.2) and phosphate buffer (PB) (pH 7.4) at 37 °C for 1 hr, 3 hr, 5 hr, 24 hr, 72 hrs. At indicated time points, nanoparticles were centrifuged at 9000 rpm for 30 min. and supernatants were collected for the estimation of peptide release by Bradford assay^57^. At the same time points, AuNPs and Pep-H-AuNPs pellets were also collected and resuspended in 2 ml of MilliQ water to record the spectra by UV-Vis spectroscopy.

### Uptake of Pep-H-AuNPs by MDMs

The uptake of AuNPs was studied by Inductively Coupled Plasma Mass Spectrometry (ICP MS) analysis^41^. Briefly, monolayers of MDMs were treated with AuNPs/Pep-H-AuNPs for 24 hrs at 37 °C, 5% CO_2_ in incubator. The monolayers were de-adhered using sterile cold PBS. For sample digestion, aqua regia was prepared by mixing high purity HNO_3_ and HCl in the ratio of 1:3. Each sample was digested with 0.5 ml aqua regia for 1 hr and then diluted to 10 ml with milliQ water. Different gold standards (0 ppb–20 ppb) were prepared from 100 ppb stock solution in MilliQ water along with addition of 0.5 ml aqua regia. The standards and samples were subjected to inductively coupled plasma mass spectrometer (Agilent technologies 7700X) to estimate the gold content inside the cells. Simultaneously, cell lysates were also prepared for the quantitation of protein by BCA and the amount of gold uptake by MDMs was expressed as amount of gold per unit mass of protein.

### MTT assay

The safety profile of Pep-H, Pep-H-CSNPs and Pep-H-AuNPs was monitored by change in cell viability using MTT assay^58^. The monolayers of MDMs were incubated with different concentrations (1 μg/ml–100 μg/ml) of Pep-H and its respective chitosan and gold nanoformulations at 37 °C in the presence of 5% CO_2_ for 72 hrs. In each well 110 μl of 1.2 mM MTT solution was added and incubated for 4 hrs at 37 °C. After the completion of incubation, 85 μl of supernatants from the wells were removed and to the remaining 25 μl supernatants, 50 μl of DMSO was added. The solution was mixed thoroughly to dissolve the dark blue crystals of formazan. The absorbance was read on microplate reader at 570 nm with reference at 630 nm. The control cells were taken as reference standard. Percent viability was calculated A570 of test wells/A570 of control wells x 100.

### Lactate dehydrogenase assay

The cell culture supernatants (50 μl) from the MDMs treated with Pep-H and its nanoformulations for 72 hrs were subjected to LDH assay to confirm the cell cytotoxicity. The assay was performed using Cytotox 96 assay kit (Promega) as per manufacturer’s instructions. For calculating %cytotoxicity by the peptide and its nanoformulations, a positive control of tritonX-100 leading to maximum LDH release was included. The absorbance obtained in the presence of test samples and positive control was used to calculate % cytotoxicity as A490 (Sample)/A490(Positive control) × 100.

### Hemolytic assay

Pep-H and its nanoformulations were incubated with 1% human RBCs in phosphate buffer (pH 7.4) for 1 hr and 24 hrs at 37 °C in round bottom 96 well microtiter plates. 0.1% triton X 100 and phosphate buffer were used as positive and negative control respectively. To determine haemoglobin release, supernatants were collected after centrifugation at 500 g in flat bottom 96 well plates^59^ and absorbance (A) was measured at 540 nm. The percent hemolysis was calculated as A_540_(sample) − A_540_(negative control)/A_540_(positive control) − A_540_ (negative control) × 100.

### Antimycobacterial activity of nanoformulations of Pep-H against intracellular *M. tb* H37Rv

PBMCs were isolated from blood of healthy subjects and differentiated into macrophages as described above. The antimycobacterial potential of Pep-H-CSNPs and Pep-H-AuNPs was evaluated in MDMs infected with *M. tb*. Briefly, MDMs were infected with *M. tb* H37Rv as described above and infected MDMs were treated with Pep-H-CSNPs and Pep-H-AuNPs at different concentrations (0.5 µg/ml, 1 µg/ml, 2 µg/ml, 5 µg/ml). At the end of 72 hrs incubation, lysates of monolayers were prepared and plated for counting the bacterial colonies as discussed above and results were expressed as log_10_ CFU.

### Statistical analysis

All graphical representations and statistical analysis was done by Graphpad prism software version 7.0 (trial version). All the experiments were repeated three times unless indicated otherwise. The data is represented as Mean ± SD and t-test and one-way ANOVA were performed. The p-value ≤ 0.05 was considered significant.
